# Inference of sigma factor controlled networks by using numerical modeling applied to microarray time series data of the germinating prokaryote

**DOI:** 10.1093/nar/gkt917

**Published:** 2013-10-23

**Authors:** Eva Strakova, Alice Zikova, Jiri Vohradsky

**Affiliations:** Laboratory of Bioinformatics, Institute of Microbiology, Academy of Sciences of the Czech Republic, Prague 142 20, Czech Republic

## Abstract

A computational model of gene expression was applied to a novel test set of microarray time series measurements to reveal regulatory interactions between transcriptional regulators represented by 45 sigma factors and the genes expressed during germination of a prokaryote *Streptomyces coelicolor*. Using microarrays, the first 5.5 h of the process was recorded in 13 time points, which provided a database of gene expression time series on genome-wide scale. The computational modeling of the kinetic relations between the sigma factors, individual genes and genes clustered according to the similarity of their expression kinetics identified kinetically plausible sigma factor-controlled networks. Using genome sequence annotations, functional groups of genes that were predominantly controlled by specific sigma factors were identified. Using external binding data complementing the modeling approach, specific genes involved in the control of the studied process were identified and their function suggested.

## INTRODUCTION

The expansion of high-throughput techniques in recent years has increased the potential to infer new biological knowledge from existing data and has also increased the demands of computational approaches to decipher large quantitative data sets. One of the primary challenges of systems computational biology lies in inferring gene regulatory networks among genes from time series expression data. A typical source of genome-wide information represents gene expression data obtained from microarrays that can be used for inference of transcriptional control networks. In this article, we focused on identifying the potential target genes of transcription regulators using a reverse-engineering transcriptional model based on the relationship between regulator expression profiles and the expression of its target genes.

Numerous computational approaches have been used to identify regulatory interactions between genes, including ordinary and stochastic differential equations, neural networks, dynamic Bayesian networks and information theoretic- or correlation-based methods, which are reviewed in articles of Bansal *et al.* (1) or Penfold and Wild (2). Generally, the methods differ in prior knowledge requirements and experimental data.

The majority of transcriptional models (also the one used in this article) are based on the assumption that the dynamics of the regulator at the protein level is correlated with the dynamics at the transcript level (3,4); therefore, measured protein levels can be replaced with relatively straightforwardly measured transcriptome profiles. The main reason for the approximation lies in the fact that protein dynamics currently cannot be easily measured on a global scale. The approach of Gao *et al*. (5) addresses latent regulator concentration by using Gaussian process inference techniques into the transcription model. The Gaussian process, together with the regulator translation model, was then used to rank target genes on a genome-wide scale to identify potential target genes (6), and further extension of the single regulator model was conducted by involving multiple interacting regulators (7).

In bacteria, the initiation of transcription depends mainly on sigma factors, which are proteins (regulators) that are able to recognize and bind, in the form of an RNA polymerase holoenzyme, to a specific gene promoter region (target gene) and guide RNA polymerase to start transcription. Therefore, sigma factors are the essential nodes in gene regulatory networks that govern further interactions and processes in the cell. A crucial task involved in inferring gene regulatory networks in bacteria is the recognition of the target genes of sigma factors. Experimentally, the physical interaction between sigma factors and gene promoter sequences is verified by chromatin immunoprecipitation methods (ChIP-chip and ChIP-Seq). It was shown, however, that the static binding information may also include silent binding events that do not directly enhance transcription (8,9). A combination of kinetic expression data with static binding site predictions represents an advantageous approach for inferring functionally related interactions between sigma factors and target genes. The dynamic model of gene expression was used here to explore the kinetically plausible regulatory relationships between sigma factors and their potential target genes based on the newly generated time series data set mapping the germination of *Streptomyces coelicolor*.

*Streptomyces* species are Gram-positive soil bacteria that are widely studied for two primary reasons. First, they are important natural producers of diverse antibiotics and biologically active compounds. Second, due to their complex developmental life cycle (including single spore germination followed by vegetative mycelia formation, aerial hyphae growth and unigenomic spore formation) *Streptomyces* serve as model organisms for fundamental cell development studies.

During dormancy, spore content is protected against unfavorable conditions by a complex coat structure, and the metabolic activity of the cell is minimal in this life phase. The process of breaking dormancy and awakening the cell to an active metabolism is called germination. The regulation of germination is important, however, poorly understood area of *Streptomyces* biology (10–13). For systems studies, the transition from dormancy to vegetative growth represents an excellent model process due to a well-defined initial state, when the development of the system always begins from the consistent pool of protein and RNA molecules.

Individual life-cycle stages are characterized by both different metabolisms and physiologies as well as by the involvement of different regulatory and signaling pathways. Numerous proteins with regulatory functions (∼12%) are predicted to exist in the *S. coelicolor* genome (14). Recent studies have also suggested an important role of regulatory RNA molecules (15,16). The *S. coelicolor* genome possesses 65 annotated sigma factors (14). Thus, in comparison with other bacteria such as *Mycoplasma genitalium* (1 sigma factor), *Escherichia coli* (7 sigma factors) or *Bacillus subtilis* (18 sigma factors), *Streptomyces* has an enormous capacity for regulation. The complexity of regulation in *Streptomyces* has fascinated researchers for decades. Current systems approaches applied on a global genomic scale, such as transcriptomics and chromatin immunoprecipitation methods (17–21), contribute to the unraveling of this regulatory complexity.

Only a small number of *S. coelicolor* sigma factors have been functionally characterized. For example, the principal sigma factor HrdB (SCO5820) represents the primary housekeeping regulator. Similar to the primary sigma factor and also closely related in promoter recognition are three sigma factors, HrdA (SCO2465), HrdC (SCO0895) and HrdD (SCO3202); however, these three factors have been reported to be non-essential for exponential growth (22). SigB (SCO0600) and SigH (SCO5243) play important roles in the osmotic stress response (23), whereas SigH has been also suggested to influence morphological differentiation (24). SigK (SCO6520) appears to negatively control development and antibiotic production (25). Other developmental sigma factors involved in differentiation are SigF (SCO4035), the late sporulation gene that affects spore maturation (26); SigN, which is believed to control aerial hyphae composition (27); WhiG (SCO5621), which is involved in sporulation by initiating the *whi* gene cascade (28); and BldN (SCO3323), which has been suggested to participate in sporulation control (29). SigT (SCO3892) may negatively influence differentiation and secondary metabolism (30). SigE (SCO3356) was suggested to be an important regulator of cell wall biosynthesis (31). The sigma factor SigR (SCO5216) was studied for its cell defense role against thiol-oxidative stress (32–35) and protein quality control (21,36).

Most of the mentioned functional characteristics were obtained by observing mutant phenotypes and expression under different experimental conditions; however, these methods do not usually identify molecular mechanisms or direct interactions between sigma factors and their target genes. Several studies have focused on identifying potential target genes of sigma factors using both *in vitro* and *in vivo* experiments and by examining the interaction between sigma factors and a vast variety of anti-sigma factors and anti–anti sigma factors (21,34,36–38).

In this study, we applied a numerical model of gene expression kinetics to identify potential sigma factor target genes in *S. coelicolor* wild-type expression data. The used model (39) originating from formalized recurrent neural networks was derived under the consideration that transcription is a temporal dynamic action and can be described using a system of differential equations. Further evaluation of the model parameters led to the computation of the expression profiles of target genes that were then compared with measured microarray data. We monitored quantitative changes in the transcriptome over time during *S. coelicolor* germination, thus generating a large experimental data set. We measured dynamic changes in the transcriptome at 13 time points during the initial 5.5 h of *S. coelicolor* germination using microarrays (37 microarrays in total). The resulting relationships between sigma factors and regulated genes or groups of genes were interpreted in biological manner and compared with published data.

## MATERIALS AND METHODS

### Cultivation and germination

The details regarding *S. coelicolor* A3(2) M145 spore cultivation and growth were published in our previous work (13). Briefly, spores harvested from agar plates (growth for 14 days) were germinated in liquid AM media at 37°C. Spores were activated by mechanical disruption of the outer coat, and a 10-min heat shock treatment was applied to boost synchrony. Samples for RNA isolation were collected during 5.5 h of germination in 30-min intervals. Altogether, we obtained samples at 13 time points, including samples from dormant spores.

### RNA isolation from spores

To break the cells, we used a FastPrep-24 machine (Biomedicals) where the spores were mechanically disrupted in tubes containing zirconium sand, two 4-mm glass beads, 500 µl of lysis buffer (40) [50 mM Tris–HCl (pH 8), 500 mM LiCl, 50 mM EDTA (pH 8), and 5% SDS] and 8 µl of RNAse inhibitors (Biorad).The disruption occurred in 6 rounds for 35 s while the tubes were re-chilled between each round. The samples were centrifuged at 14 000 *g* for 15 min at 4°C, and phenol-chloroform RNA extractions were performed on the supernatant twice. The RNA was precipitated overnight in ethanol and 3 M sodium acetate at −20°C. Finally, the RNA was resuspended in 50 µl RNAse-free water and 0.5 µl RNAse inhibitors, and the remaining DNA was removed using a DNAse Free kit (Ambion). The RNA was stored in water at −20°C.

### DNA microarrays and data processing

RNA quality control and gene expression levels were performed by Oxford Gene Technology (Oxford, UK) using Agilent DNA microarrays covering the entire *S. coelicolor* genome and the standard bacterial RNA amplification protocol for two-channel assays by Oxford Gene Technology.

The acquired data were LOWESS normalized and filtered for background and flag information (from Agilent documentation) in the GeneSpring software to obtain genes that were significantly expressed above background and to avoid side effects of possible cross-hybridizations. These methods reduced the number of entities on a single array from 43 888 to 25 312, which finally represented the outcome for 7115 of 7825 genes. The data discussed in this publication have been deposited in the NCBI Gene Expression Omnibus (41) and are accessible using the GEO Series accession number GSE44415 (http://www.ncbi.nlm.nih.gov/geo/query/acc.cgi?acc=GSE 44415).

#### Array normalization

The experiment included 37 arrays from 13 distinct time points of *S. coelicolor* germination. The arrays shared a common reference in the red channel (Cy5), which was a mixture of RNA samples from all examined time points. The distributions of Log2Ratio values {Log2Ratio = log2 [Sample (Cy3)/Reference (Cy5)]} from each array were centered to ensure that the medians and the median absolute deviations of all the array distributions were equal. The centering was performed by subtracting the Log2Ratio median value of the array from each Log2Ratio measurement on the array and was divided by the median absolute deviation. To eliminate array outliers, we filtered out the 0.02 quantile of the least and the most intensive Log2Ratio values. Therefore, normalized Log2Ratios were exponentiated to return the values to the original scale (normalized Ratios). This type of normalization was chosen as the distribution of the Log2Ratio among experiments, and time points did not show any dependence across samples and over time. This normalization for such kind of data distribution is commonly used (42).

#### Gene expression profile inference

Time series of relative mRNA concentrations (‘gene expression profiles’) were obtained by averaging the normalized Ratios across biological replicates at specific time points and across all the gene replicate spots on the array. Before averaging, the outliers among gene replicates at individual time points were filtered using the Q-test (for 3–9 inputs) and the Pierce test (for >10 inputs).

The filtering caused a few profiles to have no value for certain time points. These zero values were examined to determine whether they occurred between two non-zero time points. In this case, the neighboring time points were non-zero; therefore, the missing value was linearly interpolated (performed for ∼100 of 7115 profiles).

### Expression profile analysis

#### Highly expressed genes

**Figure 1.**
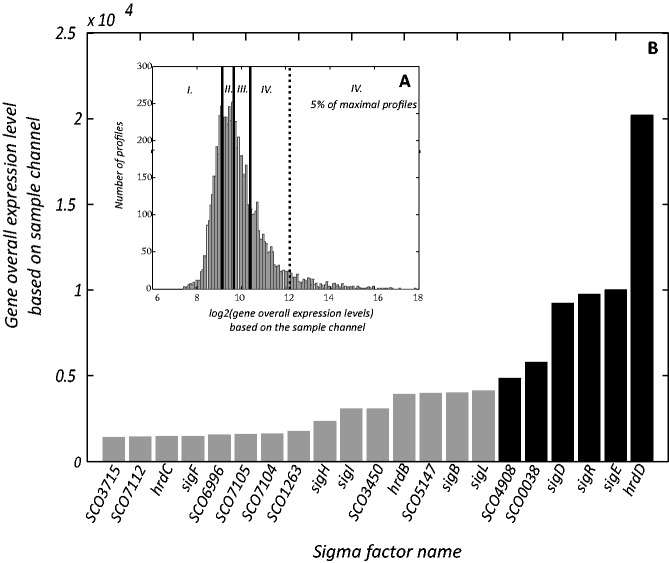
(**A**) The distribution of log2 gene overall expression levels. The black vertical lines indicate the first, second and third quartiles; the dotted line indicates 5% of the most expressed genes. The highly expressed set includes genes above the first quartile (categories II.–IV.; 75% of the data set). (**B**) The overall expression level of the 21 most highly expressed sigma factors during *S. coelicolor* germination. The gray columns represent genes with overall expression above the third quartile (category IV.), and the black columns represent genes included in 5% of the most expressed genes.

To eliminate profiles with overall low expression during germination, we analyzed microarray sample channel signals (Cy3 labeling). The idea was to minimize the influence of gene profiles whose microarray signal originated from experimental errors that exceeded the pure technical limits for eliminating signals under the background. Thus, for each gene, the overall expression level was specified by computing the median across all microarray replicates at all time points for the sample channel microarray signal. The profiles, whose overall expression level was below the first quartile value (563) of all counted medians, were filtered (category I. in Figure 1A). To avoid omitting profiles with a low overall expression level but with a significant peak, the filtered expression profiles were further confirmed. In the presence of a significant peak, the profile was considered as highly expressed and added to the set. The final set of ‘highly expressed’ genes contained 5385 profiles and was used in further analysis.

#### The selection of genes with small variance

To reduce the influence of genes with high measurement error on core profile inference, the coefficients of variation (CV) were computed for all genes at each time point. From the entire set of genes, only profiles that met the criteria of having a CV of <0.47 for at least eight time points (out of 13) in the profile were chosen. The resulting set of genes contained 3317 genes, which were further used to infer the kinetic cluster core profile.

#### Core profiles

The set of genes with a relatively small CV was grouped according to similarity in their expression profiles. The grouping was conducted in the same manner as in our previous work (13). To determine a typical kinetic profile of the particular group, we used the k-means clustering method with a Spearman correlation as a distance metric. The k-means algorithm was repeated 500 times for a predefined number of clusters (*n*). For each cluster, we then defined a cluster core as a group of profiles that appeared in one cluster in at least 50% of the repeated runs.

To roughly estimate the optimal number of clusters (*n*), the clustering procedure was recomputed for different numbers of clusters (*n* = 30–70), and a jackknife method was also applied. The jackknife was based on the systematic recomputation of clusters while omitting a small amount of random observations (1.5%). The within-cluster sums of point-to-centroid distances (J) (43) of each resulting core cluster were assessed and plotted against the number of clusters (*n*) (data not shown). The point on the curve where the significant local change of J occurred indicated the potentially optimal n for the given data set. The optimal number of clusters for the data set was *n* = 42.

For all 42 kinetic core clusters, the average profile (core profile) of each core cluster was calculated. The core profiles were then used as inputs for the modeling procedures and as the seeds for the classification of profiles into 42 groups according to their correlation with the core profile.

### Model of gene expression

We used the kinetic model of gene expression suggested by Vohradsky (39), which was later revised and extended (44,45). The model is derived from the assumption that the actual concentration of a regulator (sigma factor in this study) determines the expression kinetics of a transcribed gene. Generally, the possibility of triggering gene transcription depends on the likelihood of sigma factor binding (conjugated in RNA polymerase holoenzyme) to the promoter region. The binding probability of the sigma factor is determined by the ‘binding strength’ of the specific sigma factor to the specific gene promoter and the number of sigma factor molecules around the DNA, which is proportional to the total amount of sigma factors. When the number of regulator molecules is low, the overall regulatory effect on gene expression is small. When the number of regulator molecules increases to a certain level, the transcription rate increases and the regulatory effect on the gene expression rate is proportionally modulated by the amount of regulator molecules. The process alters until the promoter becomes saturated and an increasing number of regulator molecules do not increase the expression rate. The corresponding mathematical terms of the described relationship between the sigma factor concentration and gene expression rates represent a sigmoid function. Under previous considerations, the model for transcription control has the following form:
(1)
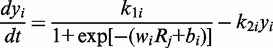

where *y_i_* represents the transcript concentration of the regulated *i-th* gene, and *R_j_* is the transcript concentration of the *j-th* sigma factor (regulator), which is modulated by parameter *w_i_*. The *b_i_* parameter corresponds to a reaction delay. Incremental expression is diminished by the rate of transcript degradation described by the term *k_2i_y_i_*. The *k_1i_*, *k_2i_*, *b_i_* and *w_i_* model parameters are derived from experimental expression data, specifically microarray data in this study.

#### Parameter optimization

To fit measured gene expression time series 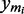 by the function y_i_, given by (Equation 1), we used an optimization procedure and computed the *k_1i_*, *k_2i_*, *b_i_* and *w_i_* parameters of the model (Equation 1)*.* For each gene, the parameters of the model (*k_1i_*, *k_2i_*, *b_i_* and *w_i_*) were optimized to fit the measured expression profile 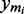 of the *i-th* gene using the measured expression profile of the *j-th* sigma factor *R_j_* by minimizing an objective function
(2)
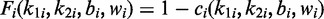

where *c_i_* represents a Pearson correlation coefficient calculated for pair 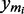 (measured expression profile) and *y_i_(t,R,k,b,w)* (computed expression profile).

Simulated annealing (46) was used as the minimization procedure. Simulated annealing performs well when the parameter space contains more local minima in which other optimization procedures can be trapped, which was considered for our data. The resulting parameters *k_1i_*, *k_2i_* and *w_i_* were forced to remain positive to reflect their biological nature.

Equation 1 was solved numerically. For numerical evaluation, the Matlab function ode45 based on an explicit Runge–Kutta formula was used.

Initial parameters were preset by random values, and then the optimization procedure was performed. For each examined relationship between sigma factor and target gene or sigma factor and another sigma factor or sigma factor and typical representative profile of kinetic cluster (core profile), the optimization procedure was completed 15 times with diverse initial parameter values. The optimal set of parameters was established as the set with the lowest value of the objective function (Equation 2); therefore, the highest correlation occurred between the modeled and measured expression profiles.

The criterion for the goodness of fit between the measured profile 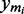 and the modeled expression curve *y_i_* was the Pearson correlation coefficient. When the correlation coefficient exceeded a predefined value, the interaction between the sigma factor and a gene was considered possible. For the modeling of regulations where no prior knowledge was available (‘Results and Discussion’), the Pearson correlation coefficient was required to be >0.8. For the modeling of interactions found by the ChIP-chip experiment or in the literature (‘Results and Discussion’), the requirement for the Pearson coefficient was arbitrarily set to 0.65 to obtain all possible and even less correlated interactions. We did not have a statistical criterion for selection of the threshold value of the coefficients, and their choice was done arbitrarily to include all possible interactions where some prior knowledge was available and after visual inspection of the results.

### Visualization of networks

The open-source software Gephi https://gephi.org/was used for network visualization.

## RESULTS AND DISCUSSION

### Experimental design

The *S. coelicolor* spores evolved during the examined period of 5.5 h from the dormant stage to cells with germ tubes.

To understand transcriptional regulations in germination in *S. coelicolor*, we used a transcriptomic-based approach in a time-dependent manner. During the monitored 5.5 h, the RNA samples were collected at 30-min intervals. The RNA sample collected from dormant spores was set as the initial time point (T Dorm), followed by RNA sample obtained after heat-shock treatment (T0) and continued by samples T0.5–T5.5 gained in 30-min intervals. Finally, we obtained RNA samples from 13 time points. For each of the 13 time points, RNA was isolated from three, for time points 4 and 7 from two, independent cultures. The mRNA expression levels were measured by microarray. In total, entire experimental set contained 37 microarrays.

### Highly expressed sigma factors in germination

The term ‘gene expression profile’ used through the text refers to the normalized Ratio signals as described in the ‘Methods’, which recorded temporal changes in mRNA expression kinetics. The arrangement of the microarray experiment (sample mRNA—Cy3 channel; reference: mixture of total mRNA—Cy5 channel) enhanced measurement accuracy but did not provide information regarding the absolute expression levels of individual genes due to the various hybridization levels of the reference caused by the diverse probes on the microarray. If we consider that an equal amount of mRNA was always loaded onto the microarray chip, we only used the sample channel signals (Cy3) to estimate the absolute expression levels of individual genes. Although this approach led to increased variance of the averaged expression values, the expression kinetics of the sample channel and kinetics of the normalized Ratios stayed highly correlated (data not shown), indicating that the overall kinetic trends were similar for both types of data. The overall expression levels based on the sample channel signals were calculated (‘Methods’) for each gene. The logarithmic distribution (based 2) of the overall expression levels was approximately lognormal (Figure 1A), with the long right tail representing ∼5% of genes with extreme overall expression in comparison with the entire data set.

Among the highly expressed genes (overall expression level above the first quartile, categories II.–IV. Figure 1A), 45 of the 65 annotated sigma factors in the *S. coelicolor* genome were detected. The overall expression levels of the most transcribed sigma factors (category IV. Figure 1A) are shown in Figure 1B, and all highly expressed sigma factors are listed in Supplementary Table S1. This article discusses the identification of regulatory interactions of these highly expressed sigma factors with individual genes and gene kinetic clusters.

### Modeling of sigma factor transcriptional control

A crucial step for transcription initiation in bacteria is the recognition and binding of the sigma factor to the gene promoter region, enabling RNA polymerase to transcribe the gene into mRNA. The *S. coelicolor* genome possesses 65 annotated sigma factors that form a broad range of sigma factor–regulated gene combinations. Cell selection of an expression program, which is governed by specific sigma factors and signaling pathways, depends on the developmental stage of the cell and external conditions. The primary task was to identify sigma factors significant for directing particular developmental processes and also identify their target genes.

For estimation of such regulatory effects, we used a kinetic transcriptional model Equation 1*.* In principle, the model tests whether for the measured expression profile of the regulator exists a set of parameters that is able to simulate the expression profile of a target gene that would fit, with good accuracy, the expression profile that was measured. The genes with a good fit between their measured expression profiles and modeled expression profiles were indicated as kinetically possible target genes of sigma factors.

The investigation of one-by-one gene regulatory interactions in the entire data set is extensively computationally demanding if all sigma factor–gene combinations have to be inspected (for 45 sigma factors and 7115 expressed genes it is 320 175 combinations). In reality, genes controlled in the same way share the same kinetic profile pattern. Instead of computing one to one interactions, it is therefore possible to compute interactions between sigma factors and characteristic gene profiles based on the kinetic profile common for a group of genes without loss of generality. With this assumption in mind, we identified kinetic clusters of genes having common expression profile and modeled the interactions on global scale between all 45 sigma factors and characteristic kinetic profiles of the clusters (‘Results and Discussion’). The relation between the kinetic clusters and operons of *S. coelicolor* is discussed in the Supplementary Note S1.

To gain more detailed insight, the individual one-by-one strategy was applied solely to identify target genes of sigma factors HrdD (‘Results and Discussion’) and SigR (‘Results and Discussion’), which were selected for the following reasons—HrdD represented the most expressed sigma factor in the experiment and for SigR we were able to incorporate static ChIP-chip binding data (21). Individually we also examined the interaction between sigma factors and their target genes that were proposed in literature (‘Results and Discussion’).

### Global kinetic analysis of time series

#### Regulatory interactions between sigma factors and groups/clusters of similarly expressed genes

To compute all the potential regulatory combinations of the 45 highly expressed sigma factors and 7115 expressed genes, we would have to analyze 320 175 sigma factor–target gene combinations. Therefore, keeping in mind that genes controlled in the same way have the same expression profile, instead of investigating one-by-one gene regulatory interactions, we analyzed the transcriptome on a global scale by working with typical kinetic trends characteristic for group/cluster of genes with ‘similar’ expression profiles that, from the kinetic point of view, may be regulated in the same way.

Each typical trend (defined by the core profile of the kinetic cluster—‘Methods’) was tested as a possible target expression profile of each of the 45 studied sigma factors. To identify the typical kinetic representatives of the target genes, we first selected a subset of gene profiles with low CV within the experimental repeats to eliminate the influence of the profiles with higher measurement errors (‘Methods’). Among the selected subset of 3317 genes with low CV, 42 different kinetic groups were identified. The core profiles were determined as an average profile of the most frequently occurring members in the particular group in the repeated runs of clustering (‘Methods’).

For each pair sigma factor–core profile, the kinetic model (Equation 1) and the optimization procedure (Equation 2) were performed to identify kinetically possible regulations. Specifically, by assigning a kinetic cluster under sigma factor transcriptional control, we assumed that all members of the cluster were controlled by this sigma factor. Obtained results are referred in Table 1, the second column.

**Table 1.** The table summarizes results obtained by global kinetic analysis of microarray time series (‘Results and Discussion’) from *S. coelicolor* germination

Regulator (sigma factor)	Suggested regulation of kinetic cluster	Suggested target sigma factor gene	Suggested regulated gene functional class
HrdA	CL 11		Biosynthesis of cofactors, carriers
	CL 14		Secondary metabolism
	CL 37		

HrdC	CL 3	*hrdD*	
	CL 5	*sigH*	
		*sigR*	

HrdD	CL 8	*hrdC*	Regulation/Defined families
		*sigR*	

SigB	CL 30	*sigL*	

SigD	CL 36	SCO0037	Amino acid biosynthesis
		SCO1564	Macromolecule synthesis
		SCO7112	Regulation/Others

SigE	CL 28	SCO1564	
		SCO7105	
		SCO7112	

SigF	CL 6	SCO0632	Extrachromosomal
	CL 22	SCO0866	
	CL 31		

SigG	CL 3	*sigK*	

SigH	CL 5		

SigI	CL 1	SCO3715	Adaptation
	CL 7	*sigT*	Energy metabolism
	CL 11		Macromolecule synthesis
			Regulation/Protein kinases
			Ribosome constituents

SigJ	CL 25		Extrachromosomal
			Transport/Binding protein

SigK	CL 15	*sigG*	Degradation of small molecules

SigR	CL 8	*hrdC*	Regulation/Defined families
		*hrdD*	
		*sigH*	

SigT	CL 1		Adaptation
	CL 13		Energy metabolism
			Regulation/Protein kinases

SCO0037	CL 28	SCO1564	Amino acid biosynthesis
	CL 36	SCO7112	Macromolecule synthesis
		*sigD*	Regulation/Others

SCO0632	CL 6	SCO0866	Extrachromosomal
	CL 22	SCO2742	
		*sigE*	
		*sigF*	

SCO0866	CL 31	SCO0632	Extrachromosomal
	CL 2	SCO2742	Not classified
	CL 16	*sigF*	
	CL 22		

SCO1263	CL 18	SCO7105	
	CL 30	*sigB*	
		*sigE*	
		*sigL*	

SCO1564	CL 26	SCO0037	Amino acid biosynthesis
	CL 28	SCO4895	Macromolecule synthesis
		SCO7112	
		*sigD*	
SCO1876		*sigN*	
		*sigR*	

SCO2742	CL 22	SCO0632	Degradation of small molecules
	CL 32		Extrachromosomal
	CL 42		

SCO3709		SCO5147	

SCO3715	CL 1	*sigI*	Adaptation
	CL 7		Energy metabolism
	CL 11		Macromolecule synthesis
			Regulation/Protein kinases
			Ribosome constituents

SCO3736	CL 35		

SCO4005	CL 14		Biosynthesis of cofactors, carriers

SCO4895	CL 26	SCO1564	Amino acid biosynthesis
		SCO7112	Macromolecule synthesis

SCO4908	CL 27		

SCO4996	CL 41		

SCO5147		SCO3709	

SCO6239	CL 3	SCO7105	
		*sigG*	

SCO7105	CL 9		Macromolecule synthesis
	CL 18		

SCO7112	CL 28	SCO0037	Amino acid biosynthesis
	CL 36	SCO1564	Macromolecule synthesis
		SCO4895	Regulation/Others
		*sigD*	

**Figure 2.**
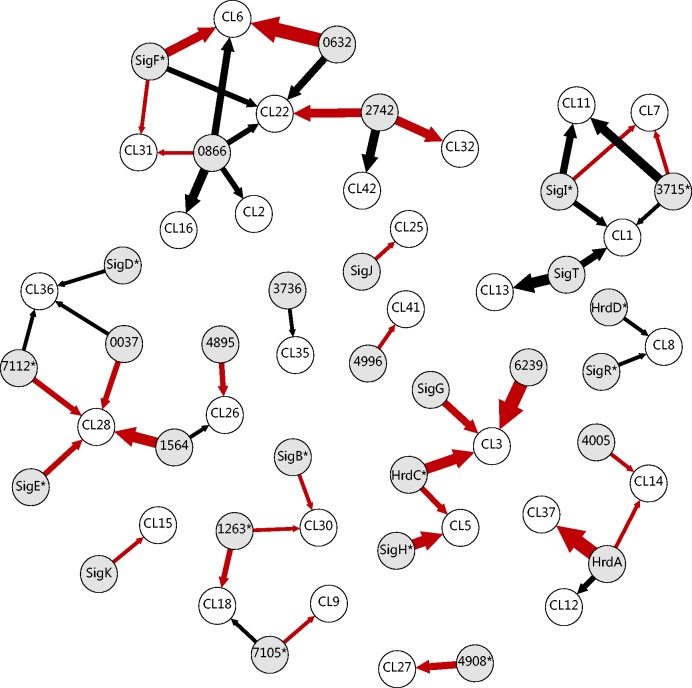
Suggested transcriptional regulation between the sigma factors (gray nodes) and the clusters/groups of genes with similar profiles (white nodes). The black arrows mark trivial regulations, whereas red arrows mark non-trivial regulations. The thickness of the arrows is proportional to parameter w of the model and corresponds to the strength of the regulatory effect. Sigma factors are marked by name or SCO number; kinetic clusters are marked with cluster number and their gene members are listed in Supplementary Table S2. Asterisks designate high overall expression of sigma factors from category IV. (Figure 1A).

**Figure 3.**
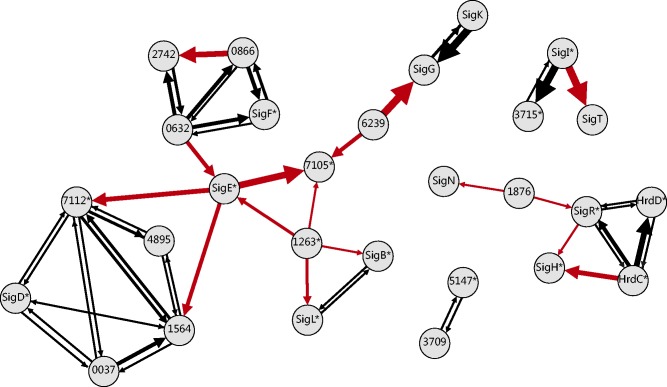
Possible transcriptional regulation between sigma factors (gray nodes). The thickness of the arrows is proportional to the parameter of the model w (Equation 1) and corresponds to the regulatory effect strength. The sigma factors are indicated by name or SCO number; those with high overall expression (above third quartile, category IV.) are marked with an asterisk. The black arrows correspond to mutual regulation (see text for details). The red arrows indicate individual regulation between regulators.

**Figure 4.**
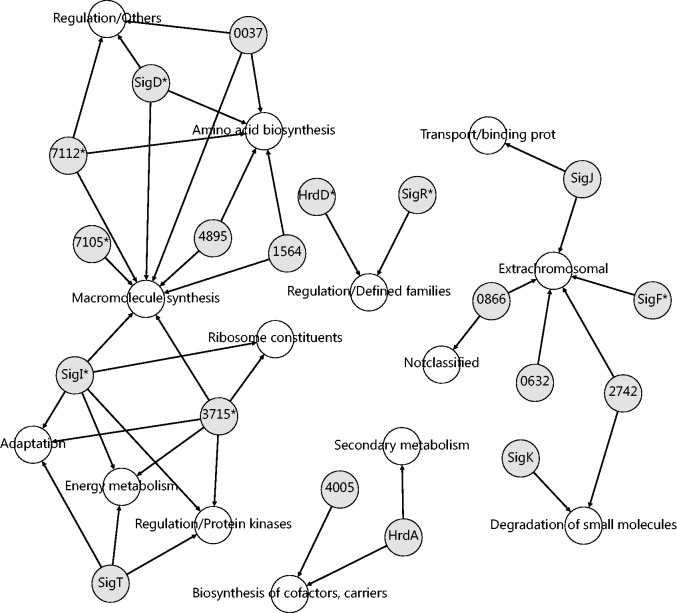
Suggested regulation of significantly enriched functional classes (white nodes) by sigma factors (gray nodes). The asterisk marks high overall expression of sigma factors from category IV. (Figure 1A).

For tested sigma factors potential regulations were suggested for: the group of genes—second column, the target sigma factor genes—third column and for the functional classes (as annotated in Sanger database)—fourth column. The individual members of kinetic clusters are listed in the Supplementary Table S2. Visualization of the second column is in Figure 2, the third column in Figure 3 and the fourth column in Figure 4.

Figure 2 presents a visual representation of the computed interactions between the sigma factors (gray nodes) and typical representative profiles of the kinetic clusters (white nodes). The thickness of an arrow corresponds to the level of influence of the regulator to the target gene and is proportional to parameter *w* of the model (Equation 1)*.* Kinetically possible regulation of a group/cluster of similarly expressed genes was found for 29 sigma factors. Remaining 16 sigma factors did not show any interactions that would satisfy the goodness of fit criteria.

A unique case occurred when the expression profile of the regulator and its target profile were highly correlated. Based on the principles of the model (Equation 1), these regulatory interactions are specific as they show minimal delay between transcription initiation and target gene expression. We will further name this type of controll as ‘trivial’. There are two possible interpretations of the trivial regulations. First, the response of the target gene to the regulator was fast; therefore, their expression profiles had a similar course. Second, the regulator and the controlled genes had a common regulator that controlled them in a similar manner. Both possibilities are equally probable, and the method used here is unable to distinguish between them. In Figure 2, the trivial regulations are marked with black arrows, whereas the other regulations are marked in red.

When more than one proposed regulatory interaction exists (more arrows are pointing to one kinetic cluster in Figure 2), it should be emphasized that all alternatives are equally probable based on the kinetic aspects and cannot be interpreted as simultaneous regulations by multiple sigma factors. Figure 2 shows all kinetically possible alternatives of regulation, and the gene members of individual kinetic clusters are listed in Supplementary Table S2.

All alternative principal sigma factors (HrdA, HrdC and HrdD), whose functions are still unknown, were suggested to control gene kinetic clusters 12, 37 (HrdA), 3 (HrdC) and 8 (HrdD). In addition to several known sigma factors (SigB, SigF, SigG, SigH, SigI and SigK) and extracytoplasmic function subfamily sigma factors (SigE, SigT and SigD), we proposed possible regulatory activity for many other sigma factors whose functions have not been previously identified (marked by SCO number in Figure 2). Detailed representation of all interactions among transcription factors and target genes is shown in Supplementary Figure S1.

#### Regulatory interactions between regulators

The same transcriptional regulatory mechanism controlling sigma factor–target gene relationships also occurs for sigma factors themselves. Thus, sigma factor expression is regulated by the interaction of either different sigma factors or autoregulated by itself. The next step of our study consisted of inspecting the potential controlling effect of a sigma factor to other sigma factors. In this case, only the expression profiles of sigma factors served as an input to the model (Equation 1).

The computed transcriptional controls between two sigma factors (listed in Table 1, the second column) are represented by arrows in Figure 3. For the measured sigma factor profiles that were highly correlated, trivial mutual interactions were found (two black arrows with opposing directions in Figure 3), which suggests that from the kinetic point of view, the regulation possibly occurred in both directions. The method used in this study did not allow us to distinguish which of them is regulator and which one is the target. The interpretation of this type of interaction is ambiguous, and further information is needed. For example, for the SigL–SigB pair, mutual regulation was proposed in this study. In the literature, SigB was suggested to be a regulator of *sigL* by Lee *et al.* (47) using different experimental approaches. Thus, by integrating the existing information, the regulation of *sigL* by SigB during *S. coelicolor* germination was much more probable compared with the SigL-*sigB* control. Incorporation of external knowledge allowed selecting the more probable of two kinetically equivalent relations. A similar case occurred for SigR–HrdD mutual regulation, where SigR was previously suggested to regulate *hrdD* (34,36) (further discussed in ‘Results and Discussion’).

#### Global functional analysis of a time series

Under the generally accepted assumption that co-expressed genes characterize a specific functional group, we examined gene members of kinetic clusters that were proposed in previous paragraphs for their membership in different metabolic groups. According to the database annotating the *S. coelicolor* genome (ftp://ftp.sanger.ac.uk/pub/S_coelicolor/classwise.txt), each gene was categorized into a functional or a potential functional class. The idea was to identify significantly overrepresented gene functional groups in the kinetic clusters, and thus characterize individual clusters. Knowing sigma factors that control the kinetic cluster, specific cell metabolic processes were characterized as controlled by individual sigma factors.

All highly expressed genes were assigned to kinetic clusters based on the value of the correlation coefficient between the given gene expression profile and the core profile of each cluster. Evidently, the choice of the criterion value may significantly affect the gene composition of the clusters; therefore, the choice of the criterion influences the functional characteristics of the group. Hence, for the enrichment of functional groups in the kinetic clusters, we tested four levels of the criterion (correlation coefficient > 0.7, 0.75, 0.8 and 0.85). Obviously, the number of genes assigned into the clusters differed with a different correlation criterion, but the changes in significantly overrepresented functional groups were minor (data not shown). Therefore, in contrast with the initial assumption, the value of the correlation coefficient was not crucial for the resulting functional characteristic of the cluster. As a selection criterion, the correlation coefficient was set to be ≥ 0.8 (3586 gene profiles assigned into kinetic clusters) (Supplementary Table S2). The coefficient can be understood as a level of membership of the gene in the particular cluster. The higher the coefficient, the more kinetically similar was the gene profile to the cluster core profile; therefore, the proposed regulation by the sigma factor had higher probability compared with genes with lower correlation coefficients.

To determine the significantly enriched gene functional groups in the kinetic clusters in comparison with the entire gene data set, statistical Fishers’ exact test was used. The functional group within the cluster was considered significantly enriched if Fishers’ test *P* < 0.05, fold-change representing the frequency of the functional group in the cluster in comparison with the frequency in entire data set > 2 and the number of genes involved in the particular functional group in the cluster was >7. The regulation of gene functional groups is depicted in Figure 4 (listed in Table 1, the third column). Gray nodes label sigma factors, whereas the white nodes represent specific gene functional groups. Similar to the case in Figures 2 and 3, multiple arrows pointing to the ‘functional nodes’ represent all controlling alternatives but not simultaneous controls.

Considerable enrichment was found for the macromolecule synthesis gene functional class, which was significantly overrepresented in four different kinetic clusters; therefore, several sigma factors were suggested to possibly regulate the group (Figure 4). Germinating spores have an urgent need for new macromolecules. It was reported (11,13) that just after germination initiation, a rapid boost of translation machinery begins. Translation is expectedly connected with ribosome constituents and amino acid biosynthesis classes of genes, which are necessary components for proteosynthesis. The other enriched gene functional groups included those associated with primary metabolism, such as genes belonging to the energy metabolism, regulations and adaptation groups.

### Biological interpretation

#### Identification of the genes controlled by SigR

As suggested in previous studies (32–35), SigR controls the oxidative stress response induced by diamide, the compound that disrupts intracellular redox homeostasis by thiol oxidation. SigR directly regulates the expression of genes that help to restore redox balance and protect the cell against chemically induced oxidations. Later studies revealed that SigR also induces target genes that participate in protein quality control, indicating that SigR regulates the cell response to protein misfolding and aggregation, also caused by the increased oxidation of enzymes induced by diamide (36). This finding is particularly important to the germination studied in this work because in dormant cells, proteins are stored as immobile aggregates (48). Further studies of the SigR regulon used the ChIP-chip assay to identify SigR binding sites under thiol-oxidative stress conditions (21), leading to suggestion of large number of SigR target genes.

From the proposed regulon of the SigR target gene (21), 145 were highly expressed in our data set. Individual kinetic profiles of this set were tested by applying the kinetic transcriptional model (Equation 1) to determine whether, from the kinetic perspective, the suggested regulation is plausible. Verification of the ChIP-chip results by using kinetic modeling was justified by the fact that the ChIP-chip assay provides only static information about SigR binding. Although binding may be observed, a functional relationship does not necessarily occur (8,9). Furthermore, several promoters can be bound by various sigma factors under different experimental conditions and developmental phases. Therefore, the verification of the kinetic plausibility of the interaction is essential.

**Figure 5.**
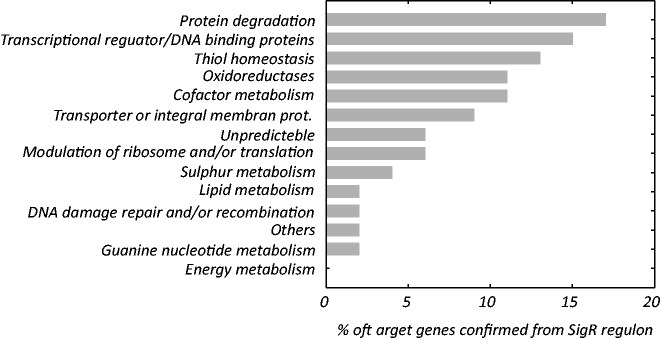
The distribution of SigR target genes that satisfied the kinetic transcriptional model among functional categories during germination. Functional assignments were adopted from the study of Kim *et al.*, 2012.

The agreement between the ChIP-chip results and kinetic modeling was observed for approximately one-third of the ChIP-chip suggested target gene set. As shown in Figure 5, the highest portions from the confirmed regulations belonged to the genes whose products have the following known/predicted function: protein degradation 17% (*clpP1*-SCO2619, *clpP2*-SCO2618, *clpC*-SCO3373, *clpX*-SCO2617, *prcA*-SCO1643, *mpa*-SCO1648 and *pepN*-SCO2643), transcriptional regulators 15% (*rsrA*-SCO5217, *ndgR*-SCO5552, *rsrA2*-SCO3451, *sigR2*-SCO3450, SCO1619 and SCO7140), thiol homeostasis 13% (*rifO*-SCO7632, *trxA*-SCO3890, *trxB*-SCO3890, *trxC*-SCO0885, *mca*-SCO4967 and *trxA4*-SCO1084), oxidoreductases 11% and cofactor metabolism 11%. Regulation for genes with other functions was confirmed for only a few (under 9%). Our approach did not confirm any regulation of genes involved in energy metabolism and identified only one target gene from the lipid metabolism group and three from the modulation of ribosomal constituents or translation group, although these groups represent 23% of target genes identified in original ChIP-chip-based approach. The full list of kinetically plausible SigR target genes in germination is shown in Supplementary Table S3. An example of the expression profiles suggested being SigR target gene is provided in Supplementary Figure S2.

Generally, kinetically confirmed SigR transcriptional controls belong to genes with ‘special’ functions in redox homeostasis, regulation or protein quality control rather than ‘basal’ metabolism functions, such as energy metabolism, lipid metabolism and ribosome constituents/translation. When interpreting the results of our experiments, the expression of SigR was not ‘arbitrarily’ enhanced by adding diamide or any other chemical compounds inducing oxidative stress, unlike in all previous studies, where the expression of SigR and its regulon was induced by diamide. Additionally, this study used cells undergoing germination; however, previous studies used cells at later life phases. When we considered that the sigma factor activity and the selection of promoters depended on growth conditions and developmental stage, the inconsistencies in our findings and the previous studies can originate from both a difference in SigR transcriptional control under ‘normal’ physiological growth conditions (germination) and under induced thiol-oxidative stress and from silent binding events.

We can conclude that during germination of *S. coelicolor*, the SigR regulon exhibited a similar response as observed under thiol-oxidative stress which has not been reported yet. We can speculate that a probable trigger of the stress that consequently induced SigR expression was a stimulus provided by high content of aggregated and/or misfolded proteins present in the dormant cell from the sporulation phase. These suggestions are in agreement with previous observations by Kalifidas *et al*. (36) where the protein misfolding and aggregation were caused by diamide resulting in an involvement of the SigR regulator in the response to the stress. In addition, the expression of several chaperones and protein modifiers, important for protein reactivation and quality control, were detected at protein level immediately after germination initiation (11,13). Here, for the first time, we were able to suggest a function of a sigma factor that acts during germination in *Streptomyces*.

#### Identification of the genes controlled by HrdD

As shown in Figure 1B, the principal sigma factor *hrdD* had the highest expression level during *S. coelicolor* germination from all sigma factors; its expression was ∼20-fold higher than the average expression level of all detected genes. Unfortunately, the specific function of HrdD in *S. coelicolor* is unknown.

Due to the *hrdD* enormous expression, we searched for potential HrdD target genes by applying the Equation 1 model in an one-by-one interaction manner to obtain more precise image of kinetically plausible HrdD targets than we reached on a global scale level (‘Results and Discussion’). In total, 88 genes met the required criterion (correlation between modeled and measured profiles >0.8). The list of these genes and their functions are shown in Supplementary Table S4. Interestingly, 30 of the 88 HrdD potential target genes were assigned a regulatory function. More than 60% of these regulatory genes belong to defined regulatory families, such as transcription regulators of the TetR, MarR, LysR and GntR families. HrdD was also found to be a potential regulator of *hrdC* and SCO0781 coding anti-sigma factor antagonist and *hrdC*. These results are consistent with the aforementioned investigation of global-scale functional analysis (‘Results and Discussion’), where HrdD was also proposed to control the overrepresented regulation/defined family’s functional group from cluster 8. Moreover, 78% of the proposed HrdD target genes also belonged to cluster 8, suggesting an agreement between global and individual gene analysis for this regulator.

Among the predicted HrdD target genes, three genes from the DnaK-HspR regulon were found; *dnaJ* (SCO3669), *hspR* (SCO3668) coding autoregulatory repressor protein and *clpB* (SCO3661) coding ATP-dependent protease) (49). More than a third of the HrdD target genes identified here belonged to a wide group of regulators. This finding suggests that HrdD may be a novel candidate sigma factor with a global regulatory role in *S. coelicolor* germination.

Among the identified HrdD target genes, several ‘trivial’ regulations (‘Results and Discussion’) were found. In the list of HrdD target genes (Supplementary Table S4), the trivial regulations are marked with a hash. For seven of these genes, the possibility of the existence of common regulators should be considered. For these genes, we previously identified possible regulation by SigR (‘Results and Discussion’), and previous binding experiments (21) also suggest that SigR is their regulator. Moreover, HrdD was suggested to be a target gene of SigR by S1 nuclease mapping and microarray experiments (34,36). In contrast, additional experiments using ChIP-chip (21) did not demonstrate the binding of SigR to the *hrdD* promoter; therefore, the interaction remains unclear. Both *hrdD* and *sigR* were among 5% of the most expressed genes during *S. coelicolor* germination and are candidates for further detailed experimental research.

#### Comparison with suggested regulations from the literature

Although the majority of *S. coelicolor* sigma factors have not been studied on a genomic scale in a systematic manner, many references mention individual genes that are regulated by sigma factors. Predicted sigma factor–target genes and the references of these studies are arranged in the ‘Database of transcriptional regulation in *Streptomyces coelicolor* and its closest relatives’ (DBSCR) http://dbscr.hgc.jp/index.html. We investigated the possibility of transcriptional regulation from the kinetic perspective, using the computational model (Equation 1) for each pair of sigma factor–target gene that was suggested by DBSRC and that was among the highly expressed genes.

Altogether, 74 regulatory relationships were tested, but only 7 satisfied the kinetic model. The verified genes are shown in Table 2 together with the list of the sigma factors and the number of tested genes identified in the literature. For SigB, the kinetic model fit for regulation of the small hydrophobic protein (SCO2372), *ssgC* (SCO7289), a *ssgA*-like gene encoding sporulation protein controlling septum site initiation and DNA segregation in spores (50) and two regulatory genes [*sigL* (SCO7278) and its putative anti-sigma factor SCO7277 (51)]. All the candidates were identified using the consensus promoter sequence and then verified by S1 nuclease mapping (51). Our computational analysis designated genes *cwgA* (SCO6179) and *dagA* (SCO3471) as target genes of SigE. CwgA is the first gene of the *cwg* operon involved in biosynthesis of cell wall glycans (52), and *dagA* is the gene that codes for extracellular agarase (53).

**Table 2.** The list of sigma factors and the number of their tested target genes based on references in the literature

Sigma factor	Gene identifier	Number of suggested target gene from the literature	Confirmed regulation for gene	Gene SCO nr.
HrdB	SCO5820	34	X	X

SigB	SCO0600	16	SCO7289	7289
			SCO7277	7277
			SCO2372	2372
			*sigL*	7278

SigE	SCO3356	9	*dagA*	3471
			*cwgA*	6179

SigH	SCO5243	8	X	X

HrdD	SCO3202	3	X	X

LitS	SCO0194	1	X	X

SigN	SCO4034	1	X	X

SigF	SCO4035	1	X	X

SigG	SCO7341	1	X	X

The references can be found at the DBSCR database webpage http://dbscr.hgc.jp/index.html.

Several factors may explain why the overlap between regulations suggested in the literature and our kinetic-based approach to be relatively low. First, the regulatory set was based on various articles (>70) describing distinct biological phenomenon under different experimental conditions and during different life phases of *S. coelicolor*. Second, numerous studies have identified the regulation of genes involved in antibiotic production or sporulation; however, these genes are highly unlikely to play an important role in germination and thus, expectedly, this regulation may not be consistent with our data set. Last, transcriptional control involves both, the binding of various sigma factors to a single promoter and the recognition of a single promoter by various sigma factors with overlapping promoter specificities (54–56). The interaction of promoter–sigma factor depends on the developmental stage and the experimental conditions. Promoter specificity overlap can be especially useful under various stress conditions that ultimately lead to the same type of physiological stress. The potential for promoter–sigma factor multiple responses also caused few genes from the tested set to be identified; therefore, in DBSRC a regulation by more sigma factors was proposed. It should be emphasized here that germination is a specific life period that may also require specific regulation and yet non-studied sigma factors.

## CONCLUSIONS

In this work, a large experimental data set containing thousands of gene temporal expression profiles that were relatively densely sampled was created. The generated data set can serve as a source material for both further computational analyses using time series expression data and consequent experimental studies.

We analyzed the whole-genome transcriptome dynamics during the transition phase of *S. coelicolor* from dormancy to vegetative growth. Using computational modeling, we identified the target genes and gene kinetic clusters of 29 sigma factors (out of 45 studied) and suggested potential transcriptional regulatory networks that are controlled by these sigma factors.

Specifically, we chose germination because it has not been studied sufficiently, although it influences further development of the cell. Germination also represents a system with a well-defined origin, which is suitable for numerical kinetic modeling applications. We showed, together with previous studies on computational kinetic modeling (2,6), that kinetic analysis of gene expression time series allows identifying gene control networks on a global scale. Although gene and protein expression levels may differ substantially, the dynamics at the transcript level is well correlated with the dynamics in protein level (3). Therefore, the time series of protein expression have been often replaced with relatively straightforwardly measured transcriptome profiles.

From the analysis of the functional groups of the target genes, we identified sigma factors that are probable regulators of basic metabolic processes activated during *S. coelicolor* germination. For a single sigma factor—SigR, the kinetic data were complemented with ChIP-chip experiment, and the results were compared with the published data. We suggest particular role for the alternative principal sigma factor, HrdD, whose expression at the mRNA level was extremely high during germination in comparison with all the other genes.

We are aware that gene expression control in *Streptomyces* is more complicated than the presented model in its current form can describe. Existence of anti-sigma factors or even anti–anti sigma factors document complex nature of the transcriptional control, making inference of the control networks in this organism complicated. In principal, two strategies for inference of gene expression control networks can be used. First, traditional, inspects sigma factors individually and experimentally searches for their targets. Such an approach has brought most of the knowledge about *Streptomyces* gene expression control available so far. However, predominantly used gene deletion and consequent pairwise comparison of mutant and wild-type strains is complicated by the inability to distinguishing between direct and transmitted control, that, to be resolved, require additional set of experiments, complicating their interpretation. The existence of >60 sigma factors in *S. coelicolor* and thousands potential target genes generate hundreds of thousands of potential regulator–target gene interactions. Their complete experimental inspection using traditional methods is virtually impossible. From such a pool, only a few were picked by other researches for detailed experimental inspections. To reconstruct the networks on a global scale from such individual results that were often obtained independently under, sometimes, different experimental conditions, is therefore difficult or even impossible, and their quantitative features from such data are completely unfeasible. A computational modeling using complex parallel kinetic data can help to overcome this hurdle, giving possibility to retain the parallel nature of the data and keep the consistency given by one experimental setup common for whole data set. Incorporating extra information such as DNA binding data to the model, as here for the case of SigR, can contribute even more to the network inference by excluding those interactions that are physically impossible and thus make the model-based predictions more accurate.

We see the contribution of this article in providing an overview of the gene expression networks active during the studied process rather than in giving ultimate answers concerning individual interactions. Our approach reduced thousands of potential regulatory interactions to tens that can be experimentally verified and gave a global outlook on the system level of control that gives a complex picture of the whole system

Last, but not least, the model presented here allows simulating kinetics of gene expression and provide possibility to make virtual experiments which can, again, point out additional experiments. Such iterative process will lead to creation of a functional model of gene expression control network that can be used to get deeper insight into the dynamic properties of gene expression control of the studied process and the topology of the network.

We are convinced that such systems level approach combining prior knowledge with computational modeling can identify, from a global perspective, regulatory networks controlling cellular processes, not only in germination and *S. coelicolor* but also in other organisms.

## SUPLEMENTARY DATA

Supplementary Data are available at NAR Online.

## FUNDING

Czech Science Foundation [P302-11-0229 to J.V.]; Grant Agency of the Charles University [17409 to E.S.]. Funding for open access charge: Publication cost grant from Academy of Sciences of the Czech Republic.

*Conflict of interest statement*. None declared.

## Supplementary Material

Supplementary Data
