# Supplementary material for: Inference of sigma factor controlled networks by using numerical modeling applied to microarray time series data of the germinating prokaryote
Source: Nucleic Acids Res. 2013 Oct 23;42(2):748–63. doi: 10.1093/nar/gkt917 (PMC3902916; doi:10.1093/nar/gkt917)
Supplement: Supplementary Data [file supp_42_2_748__index.html]

Inference of sigma factor controlled networks by using numerical modeling applied to microarray time series data of the germinating prokaryote — Inference of sigma factor controlled networks by using numerical modeling applied to microarray time series data of the germinating prokaryote — Supplementary Data 

# Inference of sigma factor controlled networks by using numerical modeling applied to microarray time series data of the germinating prokaryote

## Supplementary Data

files

**Files in this Data Supplement:**

- Supplementary Data - eps file
- Supplementary Data - eps file
- Supplementary Data - xlsx file
- Supplementary Data - docx file
