# Supplementary material for: Inference of sigma factor controlled networks by using numerical modeling applied to microarray time series data of the germinating prokaryote
Source: Nucleic Acids Res. 2013 Oct 23;42(2):748–63. doi: 10.1093/nar/gkt917 (PMC3902916; doi:10.1093/nar/gkt917)
Supplement: Supplementary Data [file supp_gkt917_nar-02411-n-2013-File010.docx]

Supplementary Note 1.

We examined the relation between gene kinetic clusters and 34 operons documented in (1). From the published set, 23 operons had all genes highly expressed in our dataset. For 15 operons, the genes were assigned to the same kinetic cluster for either all genes from the operon or more than 50% of the genes from the operon, suggesting that the regulator controlling the cluster also controls whole operon. The genes from remaining 8 operons were assigned inconsistently to different clusters.

We did not focus in more details to the association of the genes to the operon for two reasons. First, operons in *S. coelicolor* have not been extensively characterized, second, the regulation of operons seems to be more complex in *Streptomyces* as reported in Laing et al. (1) “…the control of gene expression in operons in *Streptomyces* differs from, and is more complex than that observed in *E. coli* and *B. subtilis*, and is likely to demonstrate more internal control.”, but further characterization of the type of the control hasn’t been suggested.

Supplementary note reference:

1. Laing, E., Mersinias, V., Smith, C.P. and Hubbard, S.J. (2006) Analysis of gene expression in operons of Streptomyces coelicolor. *Genome Biol*, **7**, R46.
